# Management of anaplastic thyroid cancer and proposed treatment guidelines—A 5‐year case series study

**DOI:** 10.1002/cnr2.1727

**Published:** 2022-10-04

**Authors:** Kento Koda, Mitsuhiko Katoh, Kazuo Yasuhara

**Affiliations:** ^1^ Department of Otolaryngology and Head and Neck Surgery Takeda General Hospital Aizuwakamatsu Japan; ^2^ Department of Otolaryngology and Head and Neck Surgery The University of Tokyo Tokyo Japan

**Keywords:** anaplastic thyroid cancer, radical radiation therapy, surgical oncology, surgical therapy, targeted therapy

## Abstract

**Background:**

Anaplastic thyroid cancer is a rare and rapidly progressive cancer with an extremely poor prognosis. Besides surgical control, no clear treatment has been found, mainly due to the small population affected and high mortality rate.

**Aims:**

To propose evidence‐based treatment guidelines based on a 5‐year retrospective study of patients with anaplastic thyroid cancer treated at our facility. There have been no clearly defined guidelines for treatment plan for undifferentiated thyroid cancer. Our paper presents a retrospective analysis on the treatment of patients with undifferentiated thyroid cancer at our hospital.

**Methods and Results:**

We retrospectively evaluated the data of patients diagnosed with anaplastic thyroid cancer from April 2017 to March 2022. The total number of patients diagnosed and treated was seven. Two of these patients had operable cancer; five were inoperable and treated with lenvatinib or paclitaxel maintenance therapy. The median time from the first visit to death was 3.84 months, and six of the seven patients died before this study started. Three of them had Stage IVB cancer and died due to deterioration of their general condition, including lung metastasis; the other three had Stage IVC cancer and died of suffocation. The survivor had Stage IVB cancer, was treated by surgery combined with chemical radiotherapy, and survived >240 days.

**Conclusion:**

Considering the above findings, personalized surgical treatment should be prioritized to prevent suffocation. Especially in Stage IVB cancer, local control can be achieved by surgical and anticancer drug treatment to avoid death from suffocation.

## 1 INTRODUCTION

Anaplastic thyroid cancer is a rare, rapidly progressive cancer with an extremely poor prognosis, found in ~1% of thyroid cancer cases. Besides surgical control, no clear treatment has been found, mainly due to the small population affected and high mortality rate.^1^ Japan has introduced a universal health insurance system, according to which all treatments within the insurance practice are government‐approved treatments. Treatment of undifferentiated cancer within insurance practice in Japan includes surgery, chemotherapy, and radiation therapy. However, a clear treatment algorithm has not been established, and clinical guidelines have not been unified. We performed a 5‐year case series study on patients with anaplastic thyroid cancer treated at our hospital to propose evidence‐based treatment guidelines at our facility.

## 2 METHODS

We retrospectively evaluated the medical records of all patients diagnosed with anaplastic thyroid cancer by cytology and histology from April 2017 to March 2022 at our hospital in Aizuwakamatsu, Fukushima, Japan, which is specialized in the treatment of head and neck cancer. We included all patients with anaplastic thyroid cancer who visited our hospital during the period.

There was no exclusion criteria. The total number of patients was seven.

We collected information on patient age, sex, treatment method, and survival time. Cancer was classified based on the TNM staging system, which conforms to staging according to the Union for International Cancer Control (UICC), Geneva, Switzerland.

## 3 RESULTS

We identified seven patients (four men and three women) who were diagnosed with undifferentiated thyroid cancer in the 5‐year study period (Table 1).

**TABLE 1:** Treatment results and life expectancy in undifferentiated thyroid cancer at our hospital in the last 5 years

No.	Age (years)	Sex	Survival time	First line	Second line	Third line	Cause of death	UICC ver.7 c stage		UICC ver.8 c stage		Prognostic index
1	73	M	93 days	‐	Lenvatinib	‐	Deterioration of general condition	TXN1bM0	Stage IVA	TXN1bM0	Stage IVB	1
2	75	F	>231 days	OPE	PTX 30 mg + RRT	PTX 80 mg	N/A	T4aN1bM0	Stage IVB	T2N1bM0	Stage IVB	1
3	56	M	239 days	‐	Lenvatinib	‐	Deterioration of general condition	T4bN1bM0	Stage IVB	T4N1bM0	Stage IVB	1
4	82	F	117 days	OPE	‐	‐	Deterioration of general condition	T4bN1bM0	Stage IVB	T4bN1bM0	Stage IVB	1
5	58	M	50 days	‐	Lenvatinib	‐	Suffocation	T4bNXM1	Stage IVC	T4bNXM1	Stage IVC	3
6	82	F	17 days	‐	‐	‐	Suffocation	T4bNXM1	Stage IVC	T4bNXM1	Stage IVC	4
7	71	M	190 days	‐	PTX 80 mg + PRT	‐	Suffocation	T4bNXM1	Stage IVC	T4bNXM1	Stage IVC	3

Abbreviations: N/A, not available; OPE, operation; PRT, palliative radiation therapy; PTX, paclitaxel; RRT, radical radiation therapy; Suffocation, suffocation (airway's obstruction by the bulk of tumor); UICC, Union for International Cancer Control, Geneva, Switzerland.

Surgical treatment was performed in the acute phase, and patients with Eastern Cooperative Oncology Group Performance Status 0–1 received additional postoperative radiation and chemotherapy. In addition, lenvatinib or paclitaxel (PTX) was administered for treatment in the chronic phase, depending on tumor extension and degree of vascular invasion. PTX was administered at a dose of 80 mg/mm^2^ of body surface area in cases with significant vascular invasion.

Six patients died during the study period, of whom three patients with Stage IVB cancer died due to deterioration of their general condition, including development of lung metastasis. All three patients with Stage IVC anaplastic thyroid cancer died from suffocation.

The Prognostic Index^2^ 2 was 1 for all patients with Stage IVB cancer and 3 or 4 for those with Stage IVC cancer.

**FIGURE 1:**
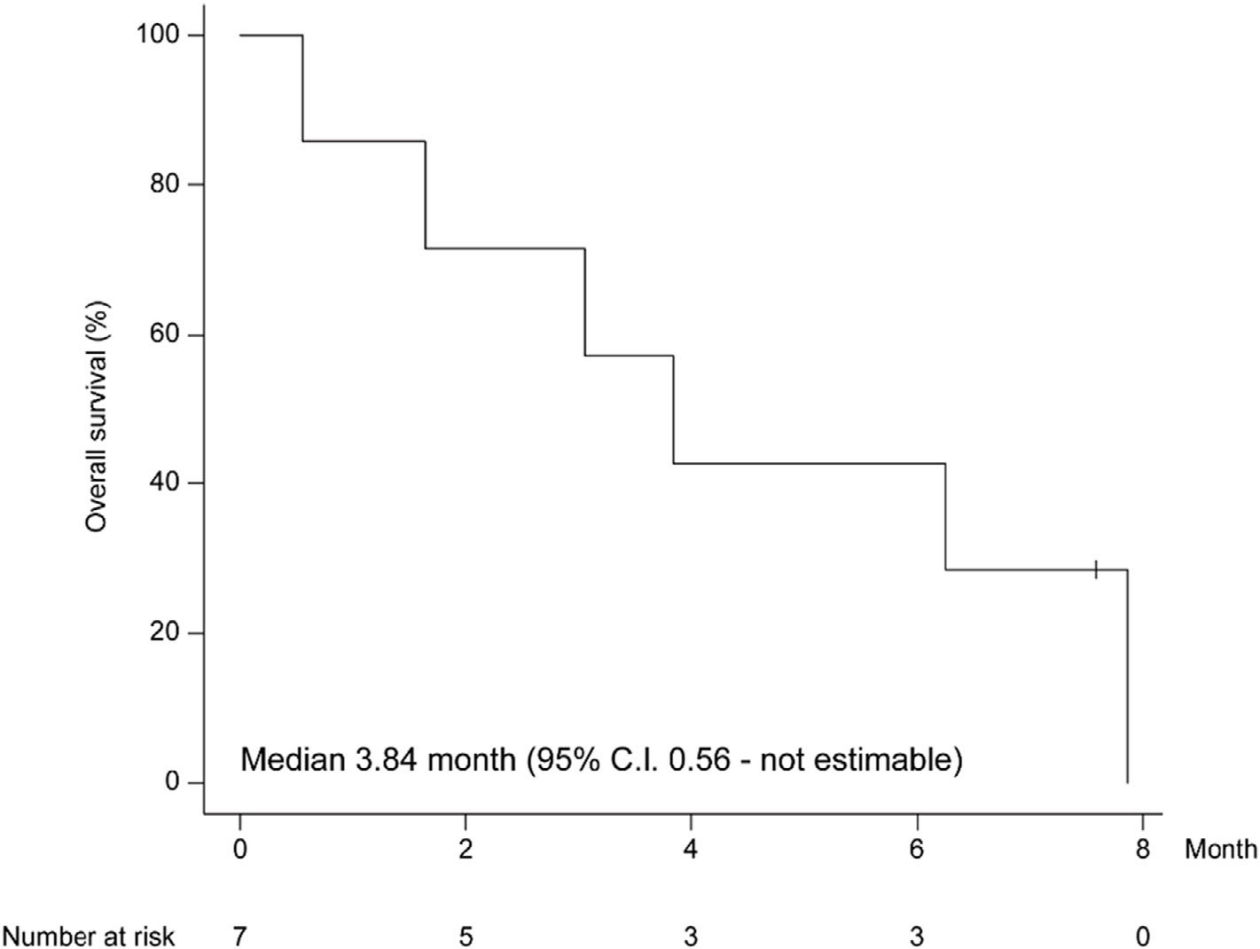
Kaplan–Meyer survival curve for patients with anaplastic thyroid cancer. CI, confidence interval

The median survival time from the initial diagnosis to death in patients with undifferentiated thyroid cancer was 3.84 months (Figure 1).

Of the seven patients, two with operable cancer were female. One developed cerebral infarction during the operation and died 4 months after being in poor general condition (Patient 4, Table 1). The other patient with operable cancer (Patient 2, Table 1) underwent total thyroidectomy and left neck dissection. During the operation, partial carotid artery wall infiltration was observed; it was sharply separated by a scalpel. We avoided commencing treatment with lenvatinib postoperatively. We considered early radiation therapy; however, there was a treatment‐free period of ~3 weeks before commencing postoperative irradiation. Hence, we administered PTX intravenously at a dose of 80 mg/mm^2^ for the first time after the wound had healed. After 1 week, 30 mg/mm^2^ of PTX and radical radiation therapy (RRT)^3^ were administered weekly for 6 weeks, as scheduled (fractionated dosing schedule for RRT included 2 Gy/day for 5 days per week from Monday to Friday).

After radiation therapy (60 Gy) completion and post‐radiation skin ulcer healing, the PTX dose was changed to 80 mg/mm^2^ weekly.^4^, ^5^ This patient was still alive for >8 months after this treatment.

Five patients with inoperable undifferentiated thyroid cancer could not receive radiation and were treated with maintenance therapy^4^, ^6^ comprising 24 mg of oral lenvatinib and 80 mg/mm^2^ of PTX administered weekly. However, all these patients died within 7 months. The cause of death was determined using imaging tests to be asphyxiation. We also determined other causes of death (Difficulty in food ingestion due to aspiration pneumonia or tumor cerebral infarction, etc.) using imaging tests. However, we do not have any explanation for the increased possibility of death by suffocation in patients with stage IV metastatic disease.

## 4 DISCUSSION

This 5‐year retrospective analysis included seven patients with undifferentiated thyroid cancer who visited a general municipal hospital. Our facility is an insurance medical treatment hospital, and the otolaryngology department does not provide any treatment outside the insurance medical treatment.

Our hospital is the core hospital in the area, and there are no large university hospitals in the neighborhood. Moreover, this area has an aging population, and many patients have difficulty visiting distant hospitals. Thus, our aim is to provide treatment that can extend survival time with less burden on patients in community medicine.

The age of onset of undifferentiated conversion was 55–75 years among men and 75–85 years among women. This is consistent with the results of two previous studies including 5^7^ and 12^8^ men in their 50s and 60s with undifferentiated thyroid cancer. These data suggest that men may be more likely to develop undifferentiated cancer younger than women. The eighth edition of the UICC states that patients with Stage IVB anaplastic thyroid cancer can survive ≥3 months with appropriate treatment, whereas those with Stage IVC survive <3 months. Herein, we present a patient (Patient 7, Table 1) with Stage IVC cancer in whom anticancer drug treatment was successful and aim to elucidate the reasons for this patient's survival in the future.

**FIGURE 2:**
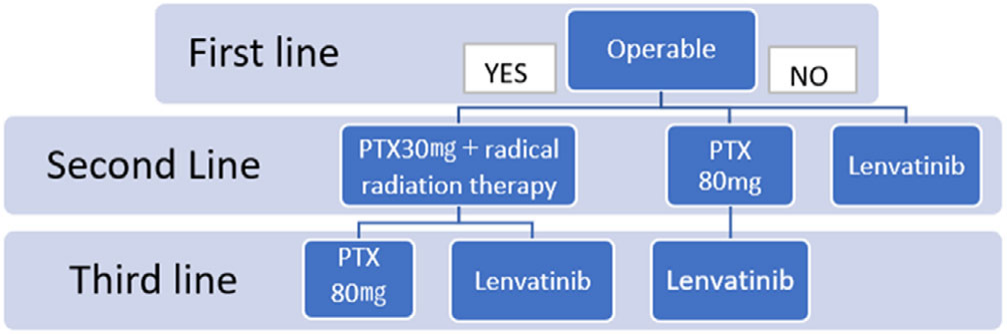
Treatment flowchart for undifferentiated thyroid cancer. PTX, paclitaxel

We suggest a new treatment algorithm for anaplastic thyroid cancer according to our evaluation of the treatment provided to the seven patients presented in this study (Figure 2). The first line of treatment for patients with undifferentiated thyroid cancer is surgery for local control purposes. If an operation is performed in patients with Stage IVB undifferentiated thyroid cancer, death from suffocation can probably be avoided. Additionally, the patient survives until the general condition is deteriorated due to lung metastasis or similar conditions. In operable cases, the second line of treatment is multimodal therapy combining 30 mg/mm^2^ of PTX and RRT, followed immediately after RRT completion by the third line of treatment comprising 80 mg/mm^2^ of PTX. Lenvatinib at a dose of 24 mg can be considered for third‐line treatment when the risk of wound healing complications is low. However, hypertension and delayed wound healing due to inhibition of angiogenesis^7^ are potential side effects of lenvatinib, and surgical procedures or radiation therapy is not recommended during lenvatinib administration.

Many cases of Stage IVC undifferentiated thyroid cancer are inoperable, even for local control, and anticancer drugs are the mainstay of treatment. In these cases, either lenvatinib or 80 mg/mm^2^ of PTX^4^ is administered as second‐line treatment. Lenvatinib is often recommended because it is administered orally on an outpatient basis, greatly improving patients' quality of life. However, in recent years, the response rate of undifferentiated thyroid cancer to lenvatinib has been reported at 2.9%. Additionally, a study reported that an antitumor effect could not be expected.^9^ A synergistic relationship has been observed between lenvatinib and PTX^10^; however, this finding is still in the basic research stage.

One of the limitations of this study is the small sample size. However, the number of patients with undifferentiated thyroid cancer is small. In addition, even after including research data from 5 years, it was impossible to examine significant differences using only data from single‐facility research. Therefore, large multicenter prospective studies comparing lenvatinib and PTX are necessary.

In conclusion, based on the results of this study, we propose a new treatment guideline for patients with undifferentiated thyroid cancer. If surgery is possible, we recommend administering PTX at a dose of 30 mg/mm^2^ with RRT,^3^ followed by weekly administration of PTX at a dose of 80 mg/mm^2^ as a postoperative adjuvant therapy.^4^ Once post‐surgery or radiation wound healing is complete (evaluated using contrast‐enhanced CT for the vessels and visual examination for the complete healing of the radiation wound), lenvatinib administration should be considered.^6^ If the cancer is inoperable, weekly either 80 mg/mm^2^ doses of PTX^4^ or lenvatinib^6^ should be selected based on the patient's quality of life.

A recent review by Arnaud et al.^11^ states that the median overall survival of patients varies from 4 to 10 months after diagnosis, which is similar to our finding. According to the treatment flow chart reported by Arnaud et al., surgical treatment without debulking is effective in patients with Stage IVA, whereas aggressive care is recommended for patients with Stages IVB and IVC. We also believe that the prognosis of patients can significantly improve by the above treatment protocol. Moreover, in their review, adjuvant chemo‐radiotherapy is recommended for localized disease diagnosed after primary surgical treatment. In addition, in the event of locally advanced or metastatic disease, the prognosis is very poor. These findings are in agreement with our results.

Unfortunately, it was difficult to perform PDL1 (Programmed cell Death 1‐Ligand 1) measurement and additional immunohistochemical and molecular analyses at our hospital, where treatment is based on the insurance practice in Japan. Currently, lenvatinib is the only molecular‐targeted drug that can be used in insurance treatment for anaplastic thyroid cancer in Japan; hence, we would like to make it a topic for future research.

## AUTHOR CONTRIBUTIONS

**Kento Koda:** Conceptualization (equal); data curation (equal); investigation (equal); project administration (lead); supervision (equal); validation (lead); writing – original draft (lead); writing – review and editing (lead). **Mitsuhiko Katoh:** Data curation (equal); supervision (equal); visualization (equal). **Kazuo Yasuhara:** Conceptualization (equal); investigation (equal); methodology (equal); supervision (equal).

## CONFLICT OF INTEREST

The authors have stated explicitly that there are no conflicts of interest in connection with this article.

## ETHICS STATEMENT

This study (Research Ethics Review Receipt Number: 2022‐004D) has been approved by the Takeda General Hospital Ethics Committee. This study used opt‐out based on ethical guidelines and was exempted from consent as per local guidelines from Takeda General Hospital Ethics Committee. (http://www.takeda.or.jp/2_index/2_tiken.html).

## Data Availability

The datasets generated and/or analyzed during the current study are available from the corresponding author on reasonable request. The data are not publicly available due to privacy or ethical restrictions.
